# Changes of Ictal-Onset Epileptic Network Synchronicity in Childhood Absence Epilepsy: A Magnetoencephalography Study

**DOI:** 10.3389/fneur.2020.583267

**Published:** 2020-11-12

**Authors:** Yulei Sun, Yihan Li, Qi Shi, Caiyun Wu, Jintao Sun, Qiqi Chen, Zheng Hu, Jing Xiang, Xiaoshan Wang

**Affiliations:** ^1^Department of Neurology, The Affiliated Brain Hospital of Nanjing Medical University, Nanjing Medical University, Nanjing, China; ^2^MEG Center, The Affiliated Brain Hospital of Nanjing Medical University, Nanjing, China; ^3^Department of Neurology, Nanjing Children's Hospital, Nanjing, China; ^4^Division of Neurology, MEG Center, Cincinnati Children's Hospital Medical Center, Cincinnati, OH, United States

**Keywords:** childhood absence epilepsy, epileptic network, individual specific network, ictal spikes, synchronicity

## Abstract

**Objective:** To further understand the mechanisms underlying epileptic network and the characteristics of individual specific network, we conducted a study on brain network by magnetoencephalography (MEG) focusing on patients with childhood absence epilepsy (CAE).

**Methods:** The network connectivity of 22 patients was investigated with MEG at the source level. Network connectivity of spikes and slow waves was computed with accumulated source imaging (ASI) and correlation analysis. Time–frequency analysis was used to characterize the network changes during the ictal-onset period of each patient and the potential factors.

**Results:** We found that spectral power increased at around 1 s and distributed at 2–4 Hz in all patients. Ictal spikes simultaneously showed elevation of network connectivity, predominantly excitatory connections, when generalized firing activity spread to the overall brain. High-frequency oscillations (HFOs) were prone to detect overexcited neuronal firing in certain focal areas.

**Conclusions:** Personal network changes during ictal onset had unique features in the time range and parallel seizure rhythm uniformly in every patient. There was an important time point for generalized discharges of the epileptic network. Ictal spiking activity played an important role in the epileptic network synchronicity of childhood absence epilepsy. Frequency oscillations provided references for locating abnormal changes in neuromagnetic signals.

## Introduction

Childhood absence epilepsy (CAE) is as an idiopathic generalized epilepsy type commonly seen in childhood, which accounts for about 10–17% of pediatric epilepsy, and 4–12 years old is the age of high incidence of the disease with a higher incidence of girls than that of boys (1–3). One clinical manifestation of CAE has briefly impaired consciousness, and typical electroencephalography (EEG) features are synchronously bilateral spike-and-wave discharges (SWDs) of 3–4 Hz over the background activity (4). Previous literatures (5–11) has demonstrated that CAE comes with an activated area at the onset of a seizure, spreads to other brain areas as the seizure develops, causing epileptic discharges throughout the brain network. In a study (12) of the epileptic neuronal network, the authors synoptically recognized network nodes as neurons and the neural network as an ensemble of many interconnected neurons. Fisher et al. (13) believed that epilepsy results from abnormal synchronization enhancement of the brain network. Neuronal synchronization between brain areas may regulate the information transmission among cortical regions in the coupled spatiotemporal scales and can be essential for consciousness and cognition (14), linked to the transient conscious absence of CAE patients. Thus, generalized epilepsy has been proposed to originate from one region and be rapidly engaged in the generalized-distributing network (12). However, whether there is a universal time point from which the brain partial discharge develops into the whole brain discharge is still elusive. Furthermore, little attention was paid to the individual network characteristics of CAE patients before, and there was hardly any study on the concrete network connectivity of independent spikes and slow waves at the specific time points. Here, we proposed a corollary that generalized seizures, including CAE, have a disorganized network leading to seizures outburst, as too many interconnections between brain areas may cause an irritable state for producing synchronous generalized-discharge activities. Possibly, personalized network changes are more helpful to discover the mechanism of SWDs in CAE.

In this study, we aimed to examine the individual variation and overall consistency during the generation of CAE and distinguish spikes and slow waves. We used magnetoencephalography (MEG) to explore abnormal network changes in children with CAE, which is suitable for the study of epilepsy and other network diseases, as it provides superior temporal resolution compared with magnetic resonance imaging (MRI) and comparative precise spatial resolution (15, 16). This study was expected to reveal the mechanism of the occurrence and spread of CAE and provide a reference for clinical diagnosis and treatment.

## Materials and Methods

### Patients

In this research, we included 31 newly diagnosed CAE patients. The following criteria were used for recruitment: (1) diagnosed as typical absence seizures according to the revised 2017 International League Against Epilepsy “Classification of Epilepsies and Epileptic Syndromes” (17); (2) no <1 seizure that lasted ≥4 s with 3–4 Hz SWDs bilaterally and synchronously showed on EEG recordings; (3) normal body development and neurological examination; and (4) head movement <5 mm during MEG inspection. We excluded some patients based on these cases: (1) existence of metal things in the body, such as implanted pacemakers and cochlear devices; (2) combinations with other seizure forms or other severe diseases; and (3) dramatic head movement during MRI and MEG scanning. All of the recruited patients came from the Neurology Department of Nanjing Children's Hospital and Nanjing Brain Hospital (NBH) between November 2011 and December 2019, and we got informed consent from all patients and their parents.

### Data Recordings

All CAE patients were required to undergo MEG recordings in a magnetically shielded room at the MEG Center of NBH using a whole-head CTF MEG system with 275 channels (VSM Medical Technology Company, Canada). Simultaneous EEG measurements were performed to visually identify ictal periods. The sampling rate of EEG was 6,000 Hz, and 23 electrodes were used. Before acquiring data, three coils were attached to each patient on the left and right pre-auricular and nasion positions. Before and after each acquisition, we performed the procedure of head localization to locate the patient's head with a coordinate system of MEG. During MEG recordings, all patients were asked to stay still in a supine condition and close their eyes. An audiovisual system monitored the recording process. The MEG sampling rate was set at 6,000 Hz. For one participant, eight consecutive 2-min duration blocks were collected. They could move freely in 1-min intervals continually separated. In general, the total recording time was ~1 h, whereas several more minutes were required to obtain a batch of new recording data if the head movement was more than 5 mm. When there were no spontaneous seizures in the scanning process, patients needed to hyperventilate to provoke a seizure. Before the formal data collection, environmental noise elimination will be carried out first.

### Magnetic Resonance Imaging Scanning

After MEG records, all participants were required to undergo a T1-weighted MRI scanning with a 3.0-T scanner (Siemens, Germany) or a 1.5-T scanner (Sigma, GE, USA). To precisely gain a co-registration of the two data sets, three fiduciary marks that consisted of the positions used in MEG recordings were similarly placed in MRI locations. All MEG anatomical locations registered to MRI records were identified.

### Magnetoencephalography Data Processing

**Figure 1:**
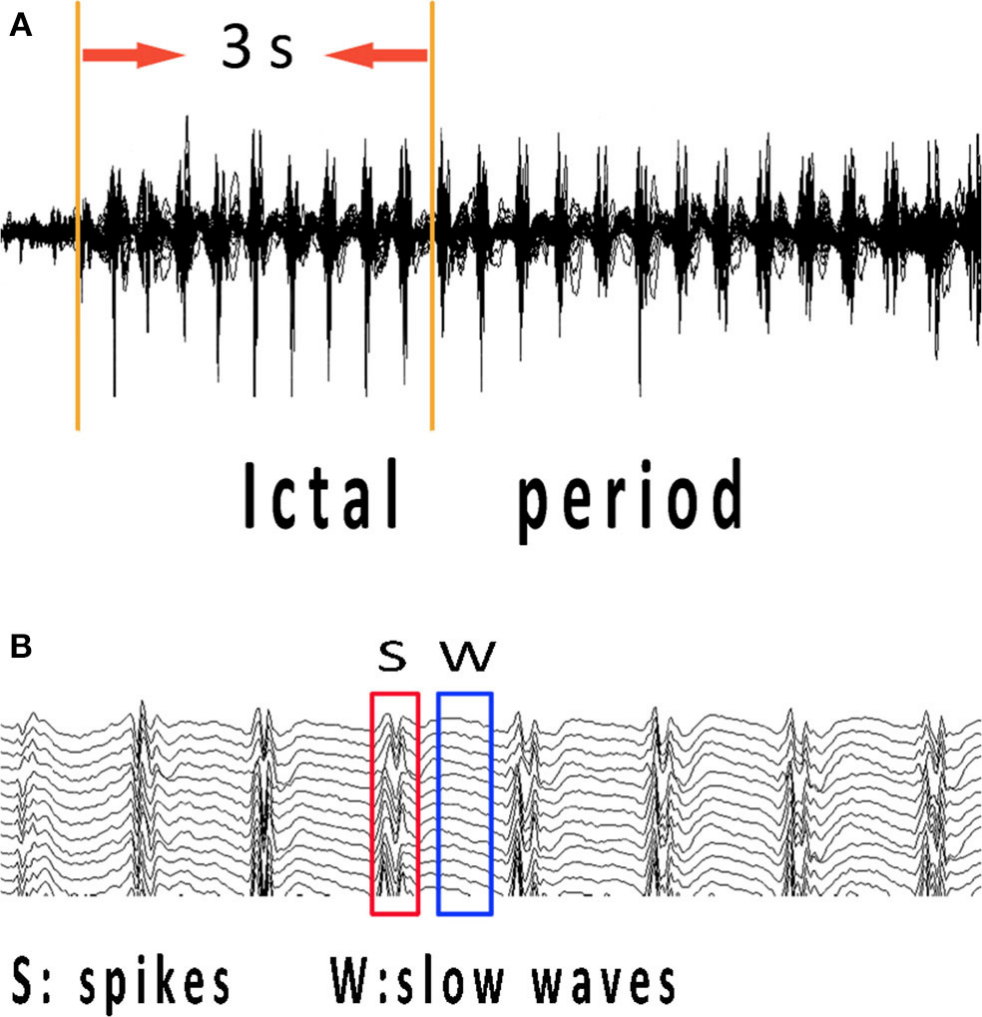
MEG recordings during ictal period of CAE. Ictal-onset 3-s was selected as research data since the first obvious spike discharge shown in **(A)**. **(B)** Independent spikes and independent slow waves at the time points corresponding to abrupt changes in spectrums of each patient were extracted separately. CAE, childhood absence epilepsy; MEG, magnetoencephalography.

The ictal onset was visually identified on EEG by an experienced neurologist using two criteria: it was significantly different from background rhythmic activities, and it could not be interpreted by other artificial or physiologic factors (18). To study the specific network characteristics in epilepsy seizures, time–frequency transform was used to analyze spectral power changes during the 3-s time interval from ictal onset (Figure 1A). The 3-s magnetic signal was filtered with the frequency band of 1–70 Hz. We selected ictal-onset 3-s because, according to the diagnostic criterion of CAE in EEG and other studies (10, 19), significant changes can be seen within at least 3 s. Then, we increased frequency bands and differentiated spikes and slow waves during this 3-s period to investigate network connectivity at specific time points. The separation of spikes and slow waves is shown in Figure 1B. The functional connectivity (FC) was used to compute the network connectivity during spikes and slow waves discharge. The numbers of total network connections were statistically compared between spike and slow-wave discharges. Further, the predominant ones in the network were analyzed between excitatory and inhibitory connections. All spikes and slow-waves data were filtered at 1–4, 4–8, 8–12, 12–30, 30–80, and 80–250 Hz, and power-line noise at 50 Hz was eliminated.

### Magnetoencephalography Data Analysis

#### Functional Connectivity Networks

FC networks were computed at the source level by accumulated source imaging (ASI) and correlation analysis. ASI was designed to sum the volumes of source activity by two-step beamforming method during a time interval and particularly analyze neuromagnetic activities in CAE patients with optimization (20–22). Firstly, we computed the source activity (or virtual sensor waveform) in the entire brain by ASI algorithms. The following equation was used for computation.

(1) $$\begin{array}{l} Asi\left(r,\;s\right)=\overset{t=n}{\underset{t=1}{\sum }}Q\left(r,t\right) \end{array}$$

*Asi* represents the source strength accumulated at point *r*; *s* indicates the time slice; *t* denotes the time point in MEG data; *n* signifies the total number of time points; *Q* is the source activity at source *r* and time point *t*. The previous articles have described the method in detail (22).

Next, correlation analysis was used to estimate the source neural networks of spikes and slow waves. We statistically analyzed the signal correlation of virtual sensors from two source pair by computing correlation factor. The correlation factor can be defined as the following formula:

(2) $$\begin{array}{l} R\left(Xa,\;Xb\right)=\frac{C\left(Xa,\;Xb\right)}{SXa,\;Xb} \end{array}$$

where *R (Xa, Xb)* represents the correlation between the location of “a” and “b” from a pair source. The *Xa* and *Xb* indicate signals in the paired sources, which are used to compute connection. *C (Xa, Xb)* represents the mean signals in the two sources. *SXa* and *SXb* indicate the standard deviation of the signals in the two sources. For avoiding possible bias, we computed all possible connections from every two-source pair by source-level analysis. All possible FC distributions of spikes and slow waves were co-registered to individual patient MRIs. Blue and red represented inhibitory and excitatory connections in MRI views, respectively.

Then, a threshold was used as a checkpoint to ensure the quality of data. T values were computed for all source pairs to determine the thresholding of connections.

(3) $$\begin{array}{l} Tp=R\sqrt{\frac{K-2}{1-R^{2}}} \end{array}$$

In Equation (3), *Tp* is the *t*-value of a correlation; *R* indicates the correlation of a source pair; *K* indicates the number of data points for connection. To obtain FC networks, we used the *Tp* value with a corresponding *p*-value <0.01 as the thresholding in the study. Lastly, FC was visually identified in three-dimensional MRI views to analyze network connectivity of spikes and slow waves. The MEG Processor (https://sites.google.com/site/braincloudx/) was used to implement the algorithms mentioned earlier, and the whole brain was scanned at a 6-mm resolution (~17,160 voxels/sources).

#### Time–Frequency Transform

In this study, the time–frequency analysis was utilized to examine the frequency and spectral characteristics in the ictal period, which was implemented with the Morlet continuous wavelet transform. The following formula can depict it:

(4) $$\begin{array}{l} G\left(t,f\right)=C_{\sigma }\pi^{-1/4}e^{{-\left(1/2\right)t}^{2}}\left(e^{i\sigma t}-k_{\sigma }\right) \end{array}$$

In this formula, *t* indicates time, and f indicates frequency. *k*_σ_ represents admissibility, and *C*_σ_ represents a normalized constant. σ represents the standard deviation of the Gaussian curve in the time domain. The related mathematical algorithms and procedures were documented in detail in previous reports (23, 24). In our research, we used real-time spectrograms to analyze the individual spectral changes in the ictal-onset 3-s period. The results were displayed in real-time spectrograms with a 512 color scale. Different color represents different power value in spectrograms. The closer the color is to red, the greater power it represents in spectrograms. The power dramatically increased occurring in red has been marked with black arrows in results.

### Statistical Analysis

Fisher's exact test was utilized to compare the differences between spikes and slow waves in network connectivity. The association between the clinical characteristics (onset age, seizure duration each time, and seizure frequency of a day) of patients and the aberrant network changes in patients were analyzed using the Pearson correlation analysis. *P*-values <0.05 of a test indicated statistically significant differences. Bonferroni correction was applied for multiple comparisons in different frequency bands. Then, we adopted the controlling procedure (20) of the false discovery rate to control the type I error. All statistical analyses and computations were performed in SPSS version 20.0 for Windows (SPSS Inc., Chicago, IL, USA).

## Results

### Patients

Twenty-two patients were involved in our study eventually, and nine patients were excluded for no seizures during scanning (even in the case of hyperventilation) or unqualified seizure data (the duration is <4 s; artificial or physiologic interference factors, like teeth grinding). Of the 22 patients, 15 patients had spontaneous absence seizures, whereas seven patients underwent hyperventilation to evoke seizures. The mean age of 22 patients was 8.52 ± 2.26 years old, with 15 females and 7 males. The seizure onset age was 7.61 ± 2.05 years old, with the minimum age of 4 years and the maximal age of 13 years old. Only seizures with duration over 4 s in MEG recordings were used in our study, and the duration recorded was from 5.50 to 35.20 s with 16.31 s on average. These patients had several seizures in a day from 1 to 15 times. Of these patients, 15 of them were drug-naïve, and the other seven had taken medicine. The relevant demographics of the patients are documented in Table 1.

**Table 1:** Demographics of patients.

**Patient**	**Sex/age (years)**	**Onset age (years)**	**Frequency (times/day)**	**Duration on recording (s)**	**Take medicine (Y/N)**	**Hyperventilate (Y/N)**
1	F/11	9	5–6	8.9	N	N
2	F/9	8	14	8.8	Y	N
3	F/6	6	10–15	12.3	Y	N
4	F/5	4	3–4	11.3	Y	N
5	M/10	9	4–5	22.7	N	N
6	F/9	8	3–4	16.3	Y	N
7	F/7	6	13–14	11.5	N	Y
8	F/9	9	2–5	8.9	N	Y
9	F/11	6	10–12	15.5	Y	N
10	M/8	7	10	35.2	N	N
11	M/10	9	4–5	32.4	N	N
12	F/10	9	5–6	10.8	N	N
13	M/14	13	2–3	12.3	Y	N
14	F/5	5	10	10.2	Y	Y
15	M/8	7	20	13.5	N	N
16	F/8	7	7–8	33.6	N	Y
17	M/5	5	7–8	12.9	N	Y
18	F/10	9	7–8	33	N	Y
19	F/9	9	1–3	8.7	N	N
20	F/10	10	5–6	16.6	N	Y
21	F/6.5	5.5	5–6	18	N	N
22	M/7	7	3–4	5.5	N	N

*F, female; M, male; Y, Yes; N, No*.

### Individually Specific Changes During Seizures

**Figure 2:**
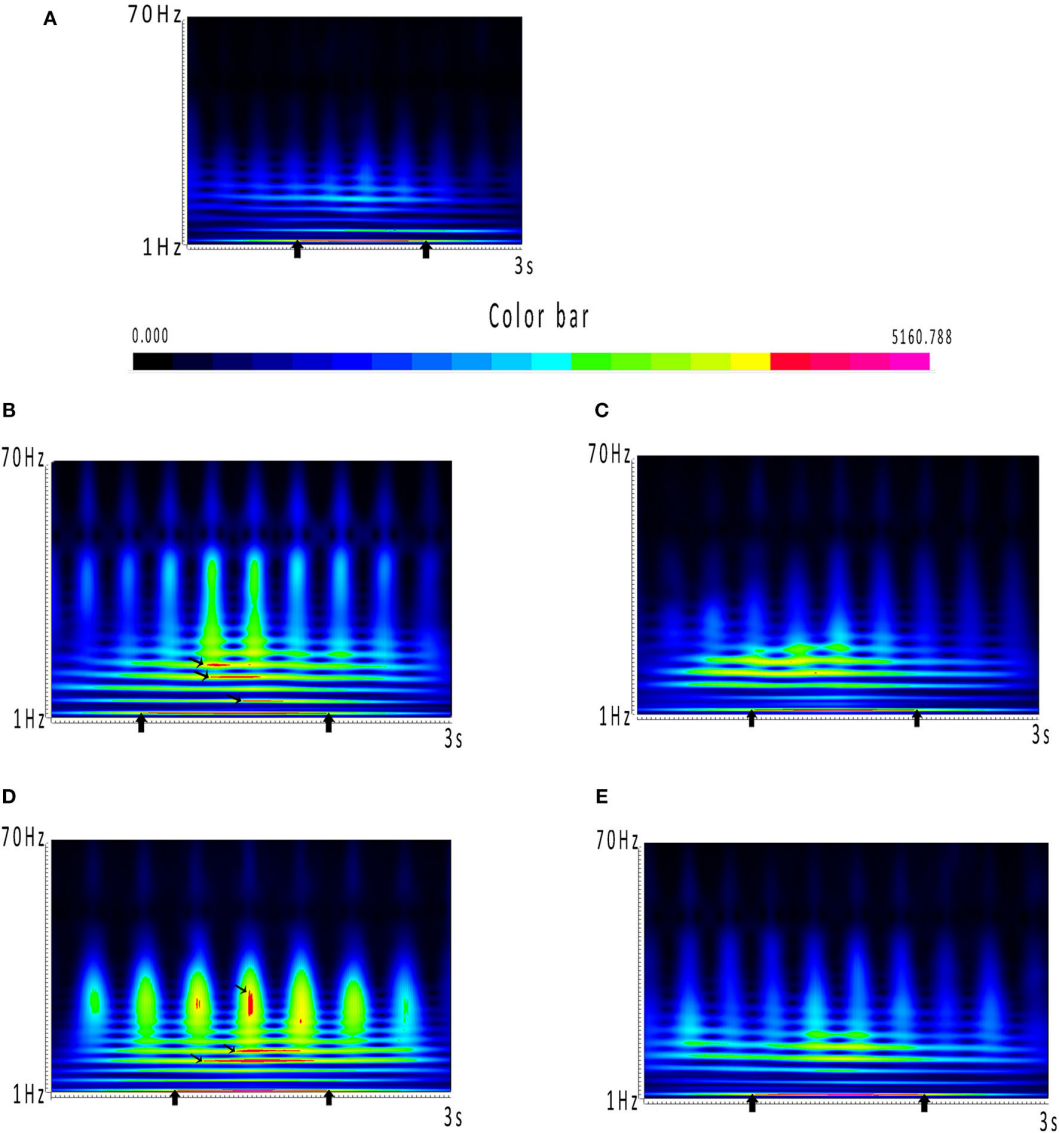
**(A–E)** Spectrums during the ictal-onset 3-s period. **(A)** Color bar of spectral power. Closer the color is to red, the greater power it represents in spectrums. Power dramatically increased occurring in red has been marked with black arrows. **(A)** Consistent result of the majority of patients, which is coming up at around 1 s and distributing at 2–4 Hz. **(B–E)** Individual spectrums distinct from the others, except from 2 to 4 Hz, which the four patients had distributions at other low-frequency bands.

The results of time–frequency transform analysis found that each participant showed distinct latency to generate spectral power explosion during the ictal-onset 3-s (Table 2). Notably, after analyzing explosion time, we concluded that all patients showed an explosion of spectral power at about 1 s from seizure onset. Among 22 patients, 14 patients started to show a spectral power explosion before 1 s, and seven patients show power change after 1 s (and the other one at 1 s). The duration of the spectral power increase of each patient exceeded 1 s and was 1.174 s on average. As for the frequency range of increased spectral power distribution, all participants uniformly came up at the frequency band of 2–4 Hz (Table 2 and Figure 2A). Apart from this consistency, four patients showed individually specific spectral changes in other low-frequency bands, including 12–14, 14–16, and 15–20 Hz (Table 2 and Figures 2B–E).

**Table 2:** Individually increased spectral power.

**Patient**	**Seizure onset (s)**	**Spectral increasing (s)**	**Increasing duration (s)**	**Main frequency distribution(Hz)**	**Other frequency distribution (Hz)**
1	80.90	81.613–82.957	1.344	3–4	6–7, 12–13, 15–16
2	0.11	1.137–2.217	1.080	2–4	-
3	31.10	32.136–33.241	1.105	2–3	-
4	22.10	23.213–24.313	1.100	2–3	-
5	65.90	66.783–67.967	1.184	3–4	9–10, 12–14, 15–20
6	0	0.950–2.053	1.103	2–3	10–11, 12–14, 23–29
7	28.30	29.160–30.347	1.187	2–4	-
8	70.60	71.572–72.787	1.215	2–3	-
9	34.30	35.383–36.547	1.164	3–4	-
10	84.80	85.848–87.016	1.168	2–3	-
11	68.40	69.400–70.627	1.227	3–4	-
12	85.70	86.593–87.920	1.327	3–4	-
13	1.90	2.970–4.070	1.100	2–3	-
14	108.60	109.437–110.659	1.222	2–4	11–13, 14–16, 17–21
15	21.50	22.487–23.603	1.116	2–3	-
16	37.80	38.780–39.883	1.103	3–4	-
17	58.50	59.443–60.580	1.137	3–4	-
18	40.80	41.737–42.900	1.163	3–4	-
19	31.80	32.823–33.963	1.14	3–4	-
20	37.40	38.323–39.707	1.384	3–4	-
21	28.30	29.160–30.347	1.187	2–4	-
22	0.08	0.903–1.987	1.084	2–3	-

*Seizure onset: visible seizure onset; Spectral increasing: power explosion in spectrums; Increasing duration: power explosion continues in spectrums; Frequency distribution: power explosion distributes in spectrums*.

### Network Connectivity Comparison Between Spikes and Slow Waves

**Figure 3:**
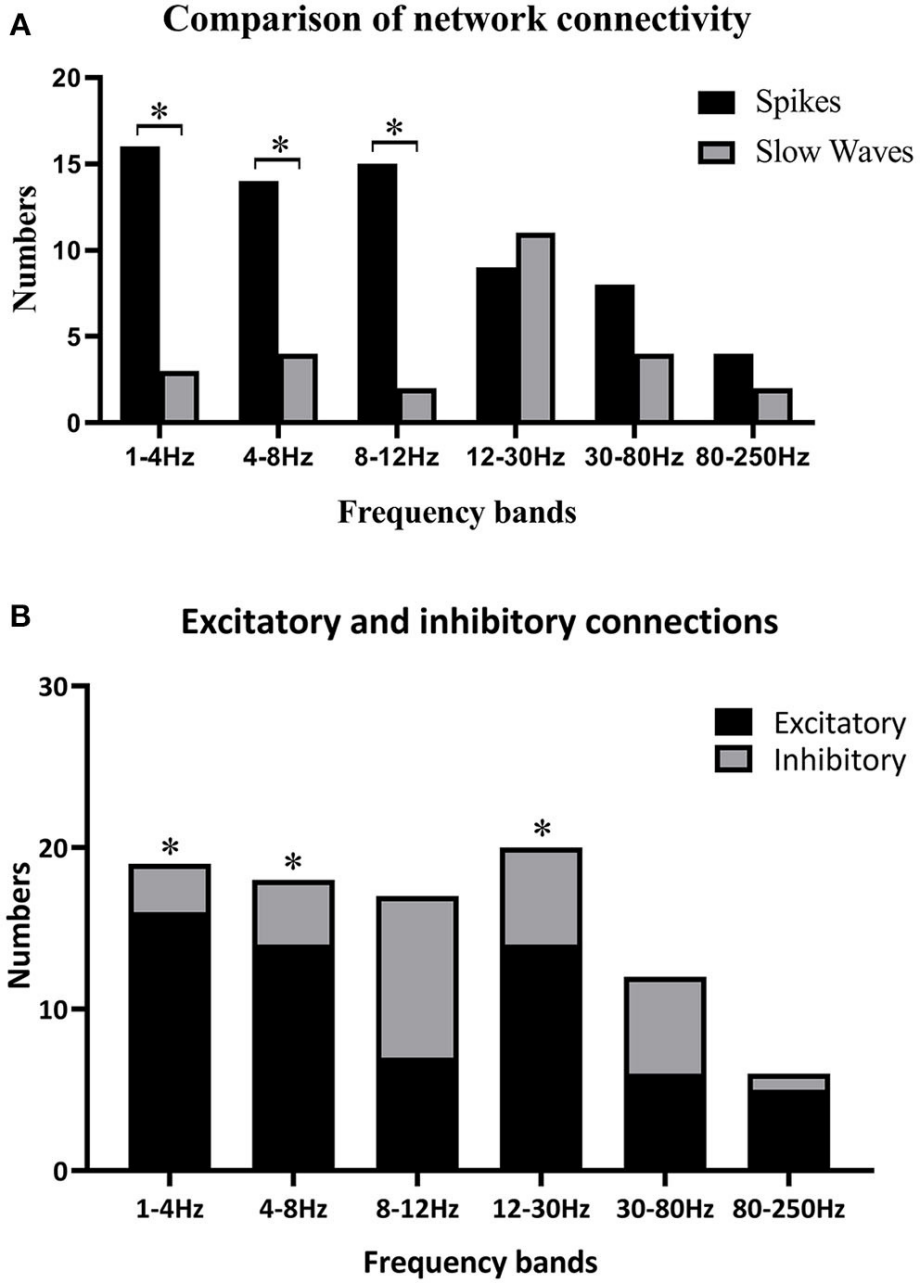
(**A)** Comparisons between spikes and slow waves in network connectivity of six frequency bands; **(B)** differences of excitatory and inhibitory connections in six frequency bands. Black asterisks are used to label some significant differences after multiple corrections when *p < 0.05. **(A)** Some significant differences between spikes and slow waves at 1–4, 4–8, and 8–12 Hz. **(B)** Excitatory connections were more obvious than inhibitory ones at 1–4 Hz (*p* ≤ 0.0001), 4–8 Hz (*p* = 0.005), and 12–30 Hz (*p* = 0.033).

**Figure 4:**
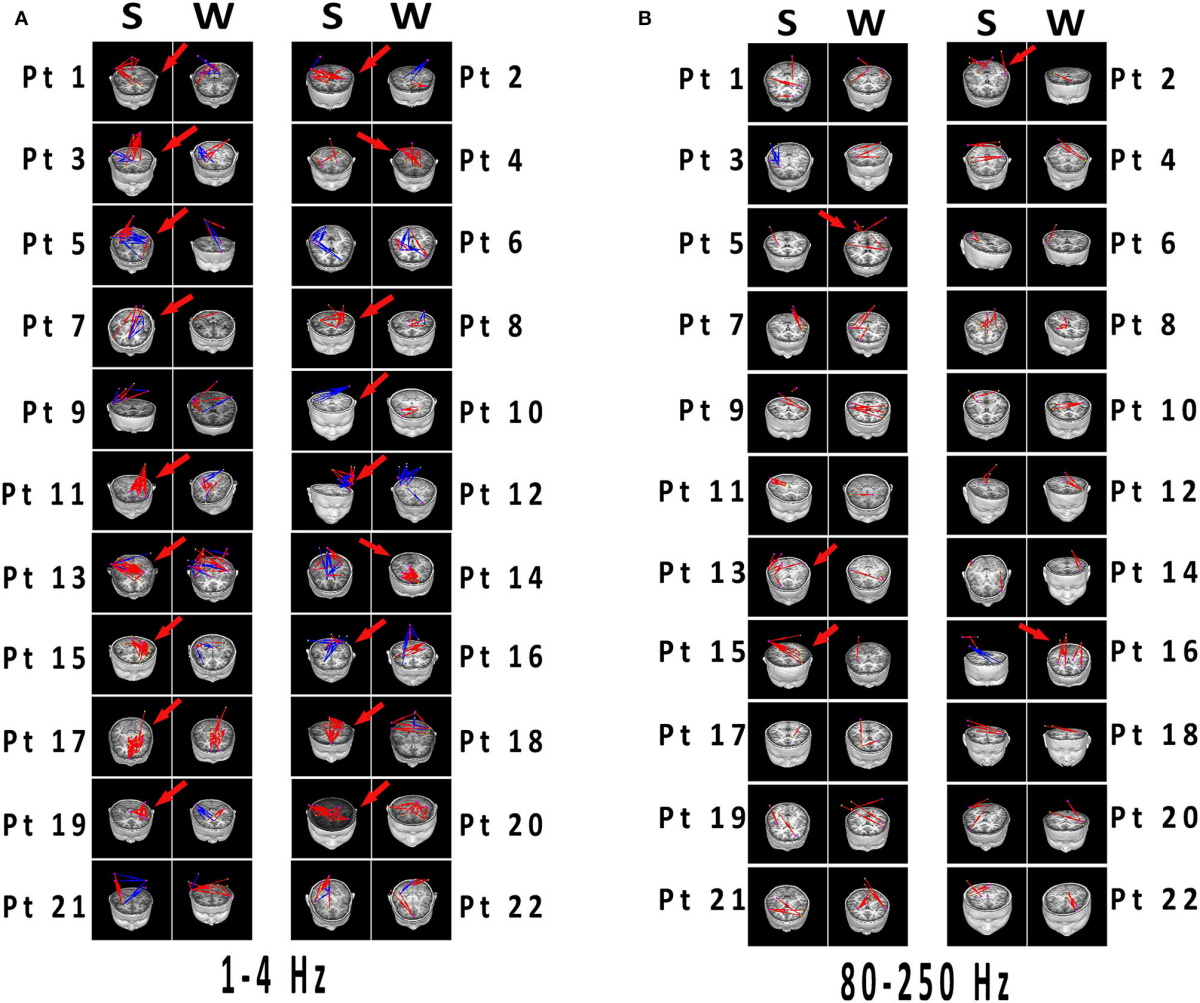
**(A,B)** Network connectivity distributions of spikes and slow waves registered to MRI. Significant differences have been labeled with red arrows. **(A)** 1–4 Hz was selected as the representation with typical distinctions, which the connections in spikes were more than that in slow waves with excitatory connections predominant. **(B)** At 80–250 Hz, both spikes and slow waves mostly showed excitatory connections. Differences were not significant in network distributions in most patients, and connections was less than that in lower frequency bands. Blue and red indicated inhibitory and excitatory connections, respectively. MRI, magnetic resonance imaging; S, spikes; W, slow waves; Pt, patient.

Because individually specific spectral power bursting always occurred in the spike discharging period, we proposed that the spike discharges could account for sudden spectral power increase. Therefore, we then compared network connectivity between spikes and slow waves. As shown in Figures 3A, 4A, at 1–12 Hz, compared with slow waves, the spikes were more likely to show synchronously increased network connections. Specifically, at 1–4 Hz, the network connection numbers of spikes were greater (16/22, *p* ≤ 0.0001), mainly due to increased excitatory connections (13/16). At 4–8 Hz, the spikes also showed increased network connections (14/22, *p* = 0.005), where the excitatory connections took up 10/14. Although spikes still displayed more network connections than slow waves (15/22, *p* ≤ 0.0001) at 8–12 Hz, the excitatory connections were not more frequently observed than those at the first two frequency bands (Figure 3B). Rather, at 8–12 Hz, the inhibitory connections played a predominant role in network synchronization (9/15). However, there were no significant differences between spikes and slow waves at the frequency bands of 12–30, 30–80, and 80–250 Hz (Figure 3A). At 12–30 Hz, spikes and slow waves were seen in 9/22 (*p* = 0.763) and 11/22 patients, respectively (two participants showed similarity in spikes and slow waves). At 30–80 Hz, spikes and slow waves were seen in 8/22 (*p* = 0.310) and 4/22 patients, respectively (10 participants had common features in spikes and slow waves). The excitatory connections were slightly more than the inhibitory connections at 12–30 and 30–80 Hz. At 80–250 Hz, it is worth mentioning that excitatory connections in both spikes and slow waves network connectivity were observed in almost all the patients, and only one patient showed complete inhibitory connections in spikes (Figure 4B). Together, these results demonstrated that the excitatory connections were more commonly seen than inhibitory connections in both spikes and slow waves network connectivity at these frequency bands. Asterisks in Figure 3B marked the statistical differences.

## Discussion

In the present study, we obtained individually specific network patterns during SWDs exploding. This can be helpful to investigate dynamic changes of CAE epileptic network. Also, we found that the special network rhythm of CAE can be traced to superabundant ictal spike discharges in brain areas. All of these results suggested that epileptic networks can play a vital role in CAE seizures.

### Individually Network Characteristics During Seizures

In our study, the increased spectral power of all patients emerged at around 1 s (Table 2). We speculated that 1 s of seizure onset is an important time point for the occurrence and development of CAE. In the previous studies, Wu et al. (25) found important source locations from preictal-2-s to onset-2-s in SWDs development and postulated the functions of the thalamus and cortex, which deepened our understanding about CAE generation. Yizhar et al. (26) used a stabilized step function opsin to test whether seizures could be prevented or aborted. They adopted a real-time detection algorithm and found that seizure onset within 1 s can block SWDs burst firing both electrically and behaviorally (26). Ossenblok et al. (27) discovered that the first generalized SWDs occurred <1 s after seizure onset in 28 patients regardless of onset age. This time point agrees with the results reported by Gupta et al. (7) that although the seizure onset age of children varied, the latency of the first SWD generation was not significantly changed. It is hard to compare these results with ours because different time windows are used and different temporal resolution of these techniques (28). Nonetheless, all these results were close to the time period of 1 s, which suggested that the duration of 1 s is essential for the strictly dynamic variations of SWDs. The present investigation will contribute to new clinical intervention and treatment for CAE patients.

What is more, the frequency band where the spectral power increased was mostly at 2–4 Hz, and four patients had increased spectral power at other low-frequency bands (Table 2 and Figures 2B–E). These results indicated that abnormal spectral changes could be detected at low-frequency bands, i.e., low-frequency oscillations (LFOs) are more sensitive to the abrupt changes of SWDs. The authors (7) chose 2–4 Hz as the frequency band computed of interest in all MEG channels with cross-spectral density matrices. Compared with the interictal periods, the power of 2–4 Hz was significantly increased locally in the frontal and occipital areas 500 ms before the first generalized spike discharge (7). The low-frequency bands have also been used in other studies (6, 20, 28–30) for epilepsy source locations or network connectivity. Interestingly, the four patients showed spectral power concentrated on other frequencies. From Table 2, the four patients had one thing in common that spectral power explosion occurred before 1 s, but what is the exact mechanism is unknown. Probably, it was attributed to individual specificity, or it could also happen to other patients, but it did not come up when we were monitoring. We did not find the relationship between network changes (the time and frequency of spectral power increasing) and clinical parameters such as onset age, seizure frequency, and duration. To verify whether there are individual-specific network changes during seizures, more efforts are needed in future experiments. We can reduce time intervals to 10 ms or even shorter and increase frequency bands (28) to test in the future to retrieve more detailed temporal information. Also, we can include the signal before and after 1 s for analysis and increase the sample size of patients to achieve a stable recording of more time points and representative dynamic evolution of the network.

### Network Connectivity Synchronization

In this part, we found that the 1-s time point was always corresponding to the spike discharges, and the network connectivity synchronization of the spike discharges was significantly enhanced, which was significantly different from that of the slow-wave discharges at 1–4, 4–8, and 8–12 Hz. These results suggested that abnormal synchronicity of brain network connectivity can be a good indicator of SWDs generation of CAE. The homeostasis systems in the brain regulate the excitability of the network, keep it in the normal range, and prevent it from saturation (31). Any slight deviation from the normal state can result in such pathological states, such as coma and seizure (32, 33). Our results found that the dynamically balanced network was distorted primarily by the increased excited connections in spikes. Some previous findings (5, 13, 34) support our results; the features of spike propagation could provide useful information about the brain epileptogenic network (5). Fisher et al. (13) found that epileptic seizures usually occur when a large ensemble of neurons was hyper-synchronously activated. The synchronization caused by neuronal spikes with oscillatory activity can lead to wide-range dynamic networks (34). The mentioned studies have suggested that the seizure of epilepsy is caused by the abnormal synchronous activity of brain neurons and might mainly relate to spike discharges, which facilitates the formation of the epileptic network. Although the exact mechanism is unclear, Sarrigiannis et al. (35) proposed that the thalamus, as a relay station in the cortical-thalamic loop, can coordinate the relationship between neuronal spike oscillations and epileptic seizures. Because there are lots of low-voltage-activated Ca2þ channels (CaV3.1, T-type) (36), the relay neurons are capable of firing paradoxical burst spiking activities after hyperpolarization of membranes (37). The subnetwork of the thalamus consists of reciprocally connected cortical-thalamic cells and reticular thalamic neurons. Also, the latter is exclusively composed of GABA-containing inhibitory neurons (38), which can also express CaV3-family channels and generate burst activities. It is proposed that the intra-thalamic excitatory/inhibitory loop can provide powerful firing, at least in coordinating the thalamic part of the network during absence seizures (39). However, more inhibitory connections appeared at alpha rhythm (8–12 Hz). CAE frequently occurred during sleeping, and alpha oscillation is close to the sleep spindles rhythm. Accordingly, we assumed that the alpha oscillation may inhibit neurons overexcited in brain networks. Nonetheless, whether there is a frequency-specific (8–12 Hz) change needs to be verified in future experiments.

Strikingly, there were almost excitatory connections at the high-frequency band (80–250Hz) regardless of spikes or slow waves. LFOs can recruit numerous neurons in broader brain areas, whereas high-frequency oscillations (HFOs) are more suitable to capture highly interconnected local and neighboring neuronal connections (40). In other words, HFOs were better to detect neuronal firing in certain focal areas, where synchronously hyper-excited neurons were populated. We concluded that HFOs are more accurate and centralized at catching neuronal firing than LFOs. The increased synchronicity of brain network connectivity during seizures is mainly due to the overactivation of excitatory neurons (Figure 3B), explaining why HFOs are basically focused on excitatory connections (Figure 4B). We found that the network connectivity strength at 80–250 Hz was not as strong as the others (Figure 4B), where the location might be the overactive neurons in the seizure zone. In many studies (40–45), HFOs, pointed as the feature of epileptic seizures, were used to locate epileptogenic zones. This is consistent with our results and speculations.

### Limitations

Certainly, some shortcomings can be seen in the present study. Firstly, the sample size was relatively small, which is not desirable to obtain more stable and consistent experimental results. Secondly, the clinical data of patients provided by their parents could be inaccurate. This may be one reason why the association between these network changes and clinical characteristics is not available. Lastly, medication might influence the results. In the future, we will consider these factors to obtain more reliable and repeatable results.

## Conclusion

Our study confirmed the existence of the CAE epileptic network, which was indispensable in seizures. Rhythmic seizure activity in the latency of 1 s was essential for the development of CAE seizures. The abnormal excitatory activity of the entire brain required a cluster of neurons of local origin to initiate spike discharges, which caused the synchronous hyper-excitability in the epileptic network. It is reasonable to believe that ictal spikes are important for spreading information from local areas to the whole brain network, which could be treated as a marker of epilepsy seizure. Frequency oscillations may provide references for locating abnormal changes in neuromagnetic signals. Taken together, our results provide the basis to explore the pathogenesis of CAE and also a new entry point for clinical intervention and treatment of the disease. More researches on individual seizure features will be carried out in the future to promote the CAE research more practical for clinical diagnosis and treatment.

## Data Availability Statement

All datasets generated for this study are included in the article/supplementary material.

## Ethics Statement

The studies involving human participants were reviewed and approved by The Affiliated Brain Hospital of Nanjing Medical University. Written informed consent to participate in this study was provided by the participants' legal guardian/next of kin. Written informed consent was obtained from the individual(s), and minor(s)' legal guardian/next of kin, for the publication of any potentially identifiable images or data included in this article.

## Author Contributions

YS designed the study and wrote this article. YS, YL, QS, CW, JS, and QC contributed to the data acquirement and analysis. ZH provided CAE patients. JX provided the MEG software. XW is the corresponding author and was mainly responsible for this project. All authors contributed to the article and approved the submitted version.

## Conflict of Interest

The authors declare that the research was conducted in the absence of any commercial or financial relationships that could be construed as a potential conflict of interest.
